# Therapeutic Potential of Clusterin Inhibition in Human Cancer

**DOI:** 10.3390/cells13080665

**Published:** 2024-04-10

**Authors:** Desirée Martín-García, Marilina García-Aranda, Maximino Redondo

**Affiliations:** 1 Surgical Specialties, Biochemistry and Immunology Department, Faculty of Medicine, University of Málaga, 29010 Málaga, Spain; desirermg@uma.es; 2 Red de Investigación en Servicios de Salud en Enfermedades Crónicas (REDISSEC), Red de Investigación en Cronicidad, Atención Primaria y Promoción de la Salud (RICAPPS), Instituto de Investigación Biomédica de Málaga (IBIMA), 29590 Málaga, Spain; marilina@uma.es; 3 Instituto de Investigación Biomédica de Málaga y Plataforma en Nanomedicina—IBIMA Plataforma BIONAND, 29590 Málaga, Spain; 4 Research and Innovation Unit, Hospital Costa del Sol, 29602 Marbella, Spain

**Keywords:** clusterin, human cancer, targeted treatment, resistance, therapeutic potential

## Abstract

Clusterin (CLU) protein is involved in various pathophysiological processes including carcinogenesis and tumor progression. In recent years, the role of the secretory isoform has been demonstrated in tumor cells, where it inhibits apoptosis and favors the acquisition of resistance to conventional treatments used to treat cancer. To determine the possible therapeutic potential of inhibiting this protein, numerous studies have been carried out in this field. In this article, we present the existing knowledge to date on the inhibition of this protein in different types of cancer and analyze the importance it could have in the development of new therapies targeted against this disease.

## 1. Introduction

Clusterin (CLU), also known by various names such as Apoliprotein J (APOJ), Complement Lysis Inhibitor (CLI), Complimented associated protein SP-40, 40 (SP-40), Sulfated Glycoprotein 2 (SGP-2), Testosterone-Repressed Prostate Message 2 (TRPM2), and Ku70-Binding Protein 1 or KUB1, is a multifunctional protein encoded by the CLU gene [1], widely expressed in various human tissues [2,3]. Over the years, CLU has been identified as a key molecule in a variety of physiological processes [3], including cell differentiation, morphogenesis [4], sperm maturation, lipid transport, complement inhibition, tissue remodeling, membrane recycling, cell–cell and cell–substrate interactions, the stabilization of stressed proteins, and cell proliferation, survival, and apoptosis [5,6].

Alterations in CLU expression have been associated with serious physiological diseases, such as spongiform encephalopathies, hippocampal and heart ischemic injuries, atherosclerosis [7], schizophrenia [8], cardiovascular diseases [9], cancer, vascular damage, diabetes, and osteoarthritis [10], and degenerative conditions such as age-related macular degeneration, retinitis pigmentosa [11], Parkinson’s disease [12], and Alzheimer’s [13,14]. These conditions are often more prominent in advanced aging [13,14], where CLU overexpression has been observed in phenomena such as enhanced cell migration [15], chemotherapy resistance [16,17], and more aggressive biological behaviors [18] in malignant cells. CLU has been proposed as a cancer biomarker [19] and cellular senescence [20], generating interest in research to better understand its implications and explore its utility in the diagnosis, prevention, and treatment of these pathologies.

## 2. Isoforms and Regulation of CLU Expression Gene

The human CLU gene, located on chromosome 8p21-p12, is a highly conserved gene with at least 63 orthologs in different species [21], and is structured with 11 exons. This gene produces at least 17 splice variants, both coding and non-coding [21,22]. However, Variant 1, Variant 2, and Variant 3 (NM-001831, NR_038335, NR_045494, respectively) are known as three transcription products that share some parts, such as exons 2-11, but differ in the first exon (1a, 1b, 1c) and untranslated terminal regions [23,24,25]. This suggests that there are different starting points for the transcription of each variant, but it is still unclear what biological function these different mRNAs have.

**Figure 1:**
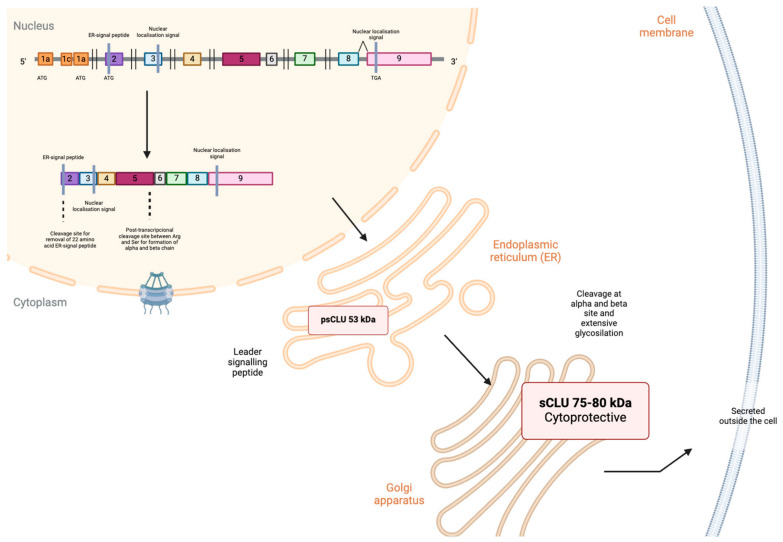
Generation of sCLU. Initially, psCLU is conveyed to the endoplasmic reticulum (ER) through the leader signaling peptide. During transit to the Golgi apparatus, it undergoes cleavage and glycosylation processes. This results in an 80 kDa protein composed of alpha and beta subunits joined by disulfide bonds, ultimately being secreted from the cell. Images were created using Biorender.com (accessed on 9 March 2024).

The synthesis of sCLU begins with the initiation codon located in exon 2 of the mRNA, leading to the formation of a preprotein comprising 449 amino acids. Initially, the sequence encodes a translocation signal that guides the preprotein to the endoplasmic reticulum (ER). In the ER, this signal is removed, and N-glycosylation takes place at six asparagine residues. This process converts the preprotein into an almost presecreted mature isoform of 53 kDa (psCLU) [26]. Subsequently, psCLU moves to the Golgi apparatus, where it undergoes further intricate glycosylation, resulting in a weight increase to between 70 and 80 kDa. The secreted isoform (sCLU), which represents the mature form of the protein, is created through the process of cleavage and the formation of disulfide bonds between residues 227 and 228 (Figure 1). Thus, sCLU is finally secreted as a heterodimeric glycoprotein complex composed of two polypeptide chains, an alpha chain (34–36 kDa) and a beta chain (36–39 kDa), connected by five disulfide bonds. It acts as an extracellular chaperone, inhibiting the aggregation of partially unfolded proteins, enhancing phagocytosis and cellular degradation, and exhibiting antiapoptotic activity in cancer cells [27,28,29]. Additionally, this variant has the capability to enhance the production of the p53 protein, which plays a role in triggering genes responsible for pausing the cell cycle, consequently inhibiting cell proliferation [3,30].

**Figure 2:**
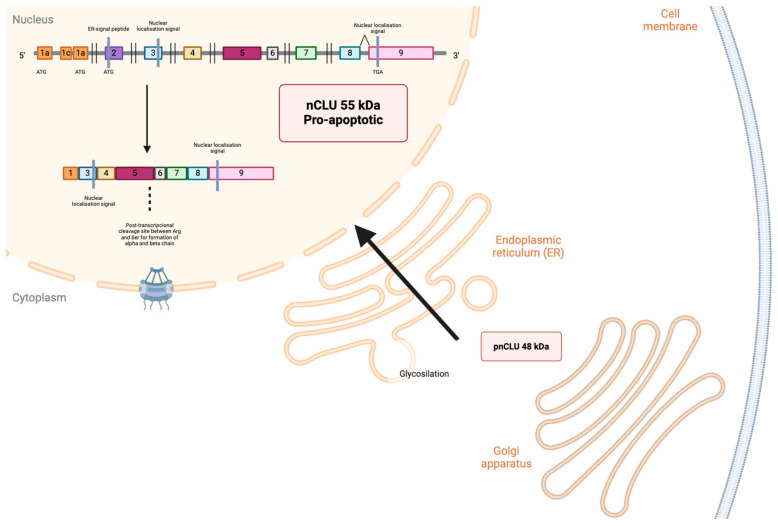
Generation of sCLU pnCLU does not undergo any excision or glycosylation process; it is localized in the cytoplasm of unstressed cells. pnCLU is converted into a mature form inside the nucleus, nCLU. Images were created using Biorender.com (accessed on 9 March 2024).

In situations of alternative splicing between exons 1 and 3, exon 2 is deleted, generating a non-functional prenuclear isoform of 49 kDa (pnCLU). In response to stress, the isoform undergoes a transformation into a functional nuclear variant known as nCLU, weighing 55 kDa. After translocating to the nucleus and binding with the Ku70 protein, it facilitates cellular apoptosis [31,32,33,34] (Figure 2).

In stressful situations, the nearly mature presecreted isoform of 53 kDa (psCLU) first interacts with the chaperone GRP78 (Bip) in the endoplasmic reticulum (ER). Subsequently, it translocates to the mitochondria, where it associates with the activated form of the Bax protein. This interaction influences the Bax protein’s ability to form homodimers and impedes the formation of Bax-Bak complexes. Within the mitochondrial, it associates with the activated form of the Bax protein, influencing its capacity to create homodimers and hindering the formation of Bax-Bak complexes [35]. This antiapoptotic influence extends to the cytoplasm, where psCLU can stabilize the Ku70-Bax complex. By doing so, it hinders Bax from reaching the mitochondria and fosters the proteasomal breakdown of cytotoxic substances [3,36].

The expression of CLU is meticulously regulated in both healthy and pathological contexts, where various pathways exert influence on its basal expression in healthy tissues as well as its upregulation in conditions such as apoptosis, pathologies, and aging. In this scenario, two forms of CLU with antagonistic functions are distinguished: sCLU, playing a cytoprotective role, and nCLU, capable of promoting cell death [37,38]. Each CLU variant is subject to different signaling pathways, depending not only on its molecular configuration but also on the cell type in which it manifests. The sophisticated regulation of both CLU isoforms encompasses a broad spectrum of factors, including growth factors such as EGF, NFGF, TGF-β, and IGF1, cytokines like IL-1, IL-6, TNF-α, and IL-2, transcription factors such as TCF1 and Jak/STAT1, as well as the Wnt signaling pathway [39,40,41,42] and epigenetic modifications. Furthermore, various factors like oxidate stress, chemotherapeutic agents, ionizing and ultraviolet radiation, estrogen, and androgen deprivation can induce their expression in hormone-sensitive tumors [43,44].

Gene expression control in vertebrates is an intricate process orchestrated by numerous regulatory proteins that collaborate to influence the expression of a specific gene or transcript within a cell. Typically, vertebrate gene expression is regulated by the basal promoter, a DNA region spanning from -200bp to the transcription start site (TSS) (+1), facilitating the binding of regulatory proteins, including transcription factors [45]. The variation in CLU expression across different tissues suggests its finely tuned and tissue-specific regulation at all levels [46]. The human CLU promoter, initially identified as a unique element, contains conserved DNA motifs, including a palindrome (-73 to -87) and a TATAA box (-26), crucial for decoding the genetic sequence. Furthermore, a GCATT box (-93) plays a key role in regulating transcription frequency [47]. Additional studies have uncovered a second promoter region (P2) within the first CLU intron, potentially controlling CLU2 expression [23]. 

In the regulation of CLU expression during aging and cancer progression, epigenetics emerges as a pivotal player, investigating heritable changes in gene expression without altering the DNA sequence [48]. The intricate interplay of DNA methylation and histone acetylation [11,49], influenced by aging and cancer, suggests epigenetic regulation’s significant role in controlling CLU [11,23,48,50].

The *Histone Code Hypothesis* outlines the diverse histone modifications governing chromatin structure and transcriptional status [51]. Histone modifications impact gene silencing or activation by modulating DNA accessibility. Notably, histone 3 lysine 9 trimethylation (H3K9me3) and histone 3 lysine 27 trimethylation (H3K27me) contribute to nCLU downregulation [48] and cell survival. Conversely, histone 3 lysine 4 trimethylation (H3K4me3) or histone 3 lysine 9 acetylation and serine 10 phosphorylation (H3K9AcS10P) lead to nCLU activation [48] and cell death. Epigenetic drug treatments alter histone modifications, impacting CLU1 and CLU2 transcription [23].

Consistent findings across diverse cell types, including cancer cells [11,23], retinal pigment epithelial cells [11], hepatocellular carcinoma cell lines [52], and endothelial tumor cells [49], emphasize the role of histone hyperacetylation induced by histone deacetylase inhibition in promoting CLU expression.

In differentiated mammalian cells, gene repression often involves DNA methylation, co-coordinated with histone modifications. While colon cancer studies indicate the predominant regulation of CLU by histone modifications [48], the presence of a G+C-rich region and a CpG mini-island within the CLU promoter suggests epigenetic control via methylation [47]. This promoter methylation is associated with decreased protein expression, observed in breast [50], ovarian epithelial cancer [18], and hormone-refractory prostate carcinoma samples [53]. Induced demethylation of CLU promoters significantly increases CLU1 and CLU2 transcripts, enhancing CLU expression across various colon cell lines [48] and tissues [54]. 

Furthermore, micro-RNAs (mi-RNAs) contribute to the post-transcriptional regulation of CLU expression [55]. In head and neck squamous cell carcinoma, oncogenic miRNA-21 specifically targets the CLU1 isoform, downregulating its growth-suppressive variant [56]. Elevated levels of miRNA-378 inhibit lung adenocarcinoma cell growth and sCLU expression [57].

While this field’s studies are recent, the emerging evidence underscores the intricate epigenetic control mechanisms orchestrating CLU expression in diverse cellular contexts. These insights provide a foundation for understanding the molecular intricacies of CLU regulation, offering potential avenues for therapeutic interventions in aging and cancer-related conditions.

## 3. Clusterin and Its Involvement in Cancer

Despite initially being believed to be linked to biological processes such as scar formation, membrane remodeling, or sperm maturation [47], early studies revealed a correlation between the overexpression of mRNA-CLU and cell death after different toxic signals [58,59,60]. However, later research presented contradictory results, establishing a relationship between CLU overexpression and cell protection [61,62].

The identification of two sets of CLU isoforms, with opposing roles and cellular locations in terms of cell survival, has contributed to clarifying these contradictions [34,63]. It has been suggested that the ratio between the expression of sCLU and nCLU is crucial in determining cancer aggressiveness. In tumor cells, a survival-favorable environment is observed with a higher sCLU/nCLU ratio, indicating elevated sCLU levels with concomitantly reduced levels of nCLU protein in the nucleus of neoplastic cells [64,65].

Post-translational modifications appear to regulate the distinctive roles of CLU, determining the expression of specific isoforms based on the presence or absence of a leader sequence in the CLU peptide [46]. Inhibition of exon 2 of mRNA-CLU, leading to the silencing of the sCLU isoform, has been crucial for the design of cancer-targeted therapies that are currently under study [66].

Early studies suggest that CLU overexpression persists after tissue degeneration, suggesting that this gene is probably not induced as a part of apoptosis that may lead to a degenerative disorder but as a secondary consequence of the disease phenotype [47]. nCLU-mediated apoptosis is associated with the accumulation of mature nCLU protein, while the rapid degradation of CLU and the regulation of its nuclear export/import can explain why highly malignant tumors avoid accumulating nCLU levels [66]. The lethality of nCLU is linked to members of the BCL-2 protein family, with nCLU-mediated apoptosis depending on Bax [64]. Additionally, nCLU can sequester the antiapoptotic BCL-XL, promoting apoptosis by forming pores in mitochondrial membranes [67].

The proapoptotic role of nCLU is also related to Ku70, which binds to CLU in response to DNA damage. The formation of a trimeric protein complex (nCLU/Ku70/Ku80) with reduced DNA end-binding activity and interaction with other proteins like Ku70 in the C-terminal coiled-coil domain of nCLU is essential for inducing apoptosis [68].

Although the constant expression of nCLU is related to cell maintenance and apoptosis induction, cells can also express cytoplasmic clusterin (cCLU) or sCLU isoforms in response to different cellular triggers. This results in increased cell survival associated with tumorigenesis, with CLU promoting apoptosis under low or moderate stress conditions but favoring the overexpression of the antiapoptotic isoform [19] under extreme cellular conditions, resulting in increased cell proliferation, viability, and invasiveness [7].

Furthermore, CLU has garnered significant attention from the scientific society because of its crucial involvement in a multitude of biological processes, including resistance to chemotherapy, cell proliferation, or apoptosis (Table 1) [69].

### 3.1. Tumorigenesis

Tumorigenesis, or tumor formation, is a complex process where normal cells transform into cancerous cells, proliferating uncontrollably. In this context, the protein CLU plays a significant role. Under normal conditions, CLU expression is low, but it significantly increases in response to stress, especially during tumorigenesis (Table 1) [72,83].

The sCLU isoform of CLU acts as an extracellular chaperone during stressful situations. This protects cells by preventing apoptosis (programmed cell death) and conferring resistance to cytotoxic agents. sCLU also plays a crucial role by interacting with protein complexes like Ku70-bax, acting as a Bax retention factor in the cytosol, inhibiting its proapoptotic function. Under normal conditions, inhibition of CLU weakens this complex, allowing Bax to translocate to the mitochondria, triggering cytochrome c release, and activating caspase 9, initiating apoptosis [70].

On the other hand, the proapoptotic isoform nCLU of CLU induces apoptosis in certain types of cancer, such as breast and prostate, through specific interactions with proteins like Ku70 and Bcl-XL [37,71].

Additionally, CLU is involved in various signaling pathways, such as B-MYB, Akt, and the PI3K/Akt pathway, regulating both apoptotic and antiapoptotic functions [84,85]. The ratio between CLU isoforms is critical for the regulation of these functions. CLU overexpression has been associated with the promotion of tumorigenesis and resistance to chemotherapy in various types of cancer, including breast and prostate [86].

### 3.2. Cell Proliferation

Cell proliferation, a cornerstone of biology, involves the proliferation of cells through repeated divisions, ensuring a dynamic equilibrium between cell death and metabolic promotion. Conversely, cell growth pertains to the physical enlargement of cell volume or size as the cell matures [87]. These processes are intricately regulated by various growth factors and cytokines in normal cells [88]. However, in cancer cells, cell proliferation becomes uncontrolled and exhibits a sustained proliferation property due to the hyperactivation of proliferative signaling pathways and evasion of growth suppressors [88].

Recent evidence highlights the involvement of the CLU protein in promoting cell proliferation and growth across different cancer types [69]. This is characterized by the acquisition of sustained proliferative signaling pathways by cancer cells (Table 1). Specifically, research indicates that the transcription factor c-Myc, encoded by the oncogene MYC and implicated in tumorigenesis, suppresses the expression of nCLU. It does so by elevating the levels of the microRNA cluster miRNA-17 ~ 92 and reducing the activity of the TGF-β axis, thus facilitating angiogenesis and tumor progression in colon cancer [73].

Both sCLU and nCLU exert notable influence on the canonical NF-κB, ERK, and AKT pathways, thereby impacting cell proliferation and growth in diverse cancer types. In prostate cancer, inhibition of sCLU with apocynin, an inhibitor of NADPH oxidase, arrests the MEK-ERK1/2 pathway, leading to suppressed cell proliferation [89]. Conversely, in osteosarcoma, induction of sCLU by DDP triggers phosphorylation of ERK1/2, stimulating cell growth and bolstering resistance to DDP [74].

The activation of the NF-κB pathway is pivotal for cell proliferation, as it prompts the expression of Bcl-2 [75]. In pancreatic cancer, melittin disrupts sCLU, thereby impeding both the cholesterol/NF-κB/Bcl-2 axis and the cholesterol/p-ERK axis, resulting in the suppression of tumor growth [75]. Interestingly, sCLU exhibits antiproliferative effects by deactivating the TAK1/NF-κB axis, preventing the recruitment of the TNF receptor-activating factor 6 (TRAF6)/TAK1-binding protein 2 (TAB2)/TGF-β-activated kinase 1 (TAK1) complex by the transforming growth factor beta receptor 1 (TGFBR1). This inhibition effectively hampers tumor proliferation and growth in human non-small cell lung cancer (NSCLC) [90].

In addition to the ERK and NF-κB pathways, the PI3K/AKT axis is also involved in regulating cell proliferation and growth. Notably, sCLU activates AKT by downregulating the expression of the protein phosphatase 2A catalytic subunit C (PP2AC) to promote the proliferation of PC-3 prostate cancer cells [91]. This aligns with the finding that silencing CLU gene transcription decreases the phosphorylation level of AKT and GSK-3β, subsequently inhibiting the proliferation of HCCLM3 cells in hepatocellular carcinoma (HCC) [92].

CLU expression and cell proliferation are influenced by various external factors. Insulin-like growth factor 1 (IGF-1), an essential component of insulin receptor signaling known for sustaining cell proliferation [81], has been found to upregulate sCLU expression. For instance, IGF-1 increases sCLU expression, activating the PI3K/AKT axis and promoting A549 cell proliferation in non-small cell lung cancer [85]. Moreover, in prostate cancer, IGF-1 enhances the transcriptional activity of the CLU gene by activating the STAT-3/Twist-1 axis. Furthermore, pituitary tumor-transforming gene (PTTG) and forkhead box protein L2 (FOXL2) influence sCLU expression and CLU gene transcription [93]. PTTG activates the ATM/IGF-1/p38MAPK/CLU axis, while FOXL2 directly binds to the CLU promoter. Interestingly, sCLU exhibits antiproliferative effects by suppressing PTTG expression, thereby restraining cell proliferation in pituitary carcinoma [76].

Metformin, a well-known medication used to treat type II diabetes, demonstrates anticancer properties. In bladder cancer, metformin works by reducing the activity of sCLU, thus inhibiting tumor growth through the deactivation of the SREBP-1c/fatty acid synthase (FASN) axis [77]. Furthermore, in renal cancer, sCLU enhances cell growth and proliferation by increasing the expression of the calcium-binding protein S100A4 [94]. In addition to metformin and melittin, compounds like epigallocatechin-3-gallate (EGCG) and green tea extract (GTE) also boost sCLU expression. They accomplish this by suppressing the activity of β-catenin, thereby promoting the proliferation of COLO 205 cells in colon cancer [95].

### 3.3. Epithelial–Mesenchymal Transition and Metastasis

Epithelial–mesenchymal transition (EMT) is a biological process involving significant changes in cell structure and function, such as loss of polarity, cytoskeletal remodeling, and acquisition of invasive properties. EMT facilitates invasion, metastasis, and tumor progression. CLU has emerged as a key regulator in these events, influencing matrix metalloproteinase (MMP) activity and the ERK1/2 and PI3K/Akt signaling pathways [96]. NF-κB activation by CLU also plays a crucial role in increasing MMP-9 and MMP-2 expression, thus promoting metastasis [79].

In different cancer types, CLU overexpression has been associated with metastasis (Table 1). In nasopharyngeal carcinoma, CLU is positively regulated by N, N′-dinitrosopiperazine (DNP), inducing MMP-9 and VEGF expression, contributing to metastasis [80]. In breast cancer, CLU collaborates with eHsp90α to activate key signaling pathways, promoting EMT, migration, and tumor metastasis [82]. In colon cancer, CLU interaction with platelets activates the p38MAPK pathway and positively regulates MMP-9, facilitating invasion [85]. Additionally, studies on prostate cancer have demonstrated that miRNA-217-5p exerts control over the processes of invasion and migration by specifically targeting CLU [97].

In patients with pancreatic ductal adenocarcinoma (PDAC), hepatocyte nuclear factor 1 b (HNF1B) has been shown to positively regulate CLU, and lower expression of both was associated with poor patient survival. Studies with pancreatic cancer cell lines have revealed that CLU inhibits cell proliferation, invasiveness, or EMT. These findings suggest that the HNF1B/CLU pathway is crucial in slowing the progression of pancreatic cancer [98].

### 3.4. Chemoresistance and Chemosensitivity with Clusterin

Chemoresistance, which involves cancer cells’ ability to withstand chemotherapy, poses a significant obstacle in cancer treatment. This phenomenon can occur through different mechanisms, including genetic alterations, changes in drug metabolism, and the suppression of apoptosis [99]. Chaperone proteins like CLU are pivotal in conferring resistance to cancer therapy, promoting the growth of malignant tumors and shielding drug-resistant cells (Table 1) [78].

The stress response triggered by treatments like radiotherapy and chemotherapy leads to increased expression of sCLU, a protective chaperone. sCLU interacts with activated Bax, inhibiting the release of cytochrome c and thus preventing apoptosis [100]. Conversely, reducing CLU levels, as observed in certain cancers like testicular seminoma, enhances sensitivity to radiotherapy and chemotherapy [101].

In hepatocellular carcinoma (HCC), sCLU is markedly overexpressed, contributing to resistance against oxaliplatin by downregulating Gadd45a expression and activating the PI3K/Akt pathway. Inhibiting CLU in HepG2/ADM HCC cells replenishes sensitivity to various drugs [102].

## 4. Clusterin as a Biomarker and Therapeutic Target in Cancer

In the field of medicine, biomarkers have become essential tools to provide valuable information about patient health and the progression of various diseases. From measuring simple protein levels to identifying genetic mutations, these markers play a crucial role in the diagnosis, prognosis, monitoring, and treatment of diseases. In this context, CLU has been studied as a potential biomarker in various types of cancer due to its tendency to be overexpressed in stressful situations (Table 2).

However, further research is needed in this field to determine the exact value of CLU levels and their association with each type of cancer. Currently, it can only be used as a tool to assess risks [140].

As a therapeutic target, the inhibition of CLU has demonstrated excellent therapeutic effects in various cancers both in vitro and in vivo, prolonging the survival of patients. Custirsen (OGX-011), a second-generation antisense oligonucleotide, has proven effective by interacting with the ATG sequence in exon 2 of the secretory isoform of CLU (sCLU), inhibiting its translation and suppressing cancer progression such as prostate cancer [15], renal cell carcinoma [141], bladder [142], liver [143], lung [144], prostate [145], breast [146], lung adenocarcinoma [104], melanoma [131], osteosarcoma [135], and ovary [147].

When combined with other cancer therapies in clinical trials, OGX 011 enhances antitumor activity. In the case of renal cell carcinoma, CLU inhibition by custirsen improves sorafenib cytotoxicity [148]. In ovarian cancer, it has been shown to improve the survival of patients treated with paclitaxel by enhancing its response [149], similar to what happens in castration-resistant prostate cancer with mitoxantrone and docetaxel by reducing sCLU expression [150,151]. Clinical studies have also explored the efficacy of custirsen in metastatic castration-resistant prostate cancer, showing promising results in early-phase trials, although the standard treatment remains prednisone and cabazitaxel [152].

In metastatic breast cancer, similar effects have been shown with combinations of OGX 011 and docetaxel [153], while in lung cancer, combinations with gemcitabine or cisplatin have extended survival [154].

Therapeutic approaches beyond OGX-011, such as RNA interference strategies including microRNA (miRNA), short hairpin RNA (shRNA), and small interfering RNA (siRNA), have demonstrated efficacy in suppressing CLU expression across various cancers. For instance, miRNA-217-5p and miRNA-195 have been found to downregulate CLU expression, impeding cell migration and invasion while fostering apoptosis in prostate cancer cell cultures [155,156]. Furthermore, several other therapeutic agents, including melittin, green tea extract (GTE), apocynin, vitamin D, metformin, and verteporfin, have exhibited antitumor properties by inhibiting CLU protein expression. Consequently, these interventions have shown promise in impeding cancer progression [75,77,89,157,158,159].

In the realm of emerging therapies, the drug AB-16B5, a monoclonal antibody targeting sCLU, is being evaluated in a phase II clinical trial in combination with docetaxel in patients with metastatic non-small cell lung cancer [160].

## 5. Conclusions

CLU plays a pivotal role in cancer progression by modulating key processes such as programmed cell death, epithelial–mesenchymal transition (EMT), metastasis, and cell proliferation and growth through intricate signaling pathways. The complexity of its biological function stems from the existence of two alternatively spliced isoforms and the variable localization of their protein products both intra- and extracellularly. Despite the intricate nature of these isoforms, the coexistence of nCLU and sCLU within cells, coupled with the precise regulation of their balance, confers either pro- or antiapoptotic properties.

Various potent inhibitors of CLU, including OGX-011 and RNA interference (RNAi) agents, have been developed for use in cancer therapies, yielding encouraging therapeutic outcomes in patients. Given the diverse regulatory pathways governed by CLU in cancer progression and the demonstrated survival benefits associated with its inhibition across different cancer types, CLU emerges as a promising therapeutic target for the development of more efficacious agents and targeted therapies. Moreover, considering its oncogenic properties, the sCLU isoform holds potential as a blood biomarker for cancer diagnosis.

However, despite extensive research on the functions of CLU isoforms, it remains unclear which isoform contributes to specific biological effects. Therefore, it is imperative to delve deeper into the distinct functional role of each CLU isoform, particularly sCLU, to refine CLU targeting as a therapeutic strategy more effectively.

## Figures and Tables

**Table 1:** Biological processes involving CLU isoforms.

Biological Processes	nCLU	sCLU
Tumorigenesis	The proapoptotic isoform nCLU of CLU induces apoptosis in breast and prostate cancer, through specific interactions with proteins like Ku70 and Bcl-XL [70,71].	sCLU also plays a crucial role by interacting with protein complexes like Ku70-bax, acting as a Bax retention factor in the cytosol, inhibiting its proapoptotic function. Under normal conditions, inhibition of CLU weakens this complex, allowing Bax to translocate to the mitochondria, triggering cytochrome c release, and activating caspase 9, initiating apoptosis [72].
Cell Proliferation	c-Myc, a transcription factor encoded by the oncogene MYC involved in tumorigenesis, inhibits the expression of nCLU by upregulating the microRNA cluster miRNA-17 ~ 92 and attenuating the TGF-β axis, thus promoting angiogenesis and tumor growth in colon cancer [69].	Blocking sCLU using apocynin, a substance that inhibits NADPH oxidase, halts the MEK-ERK1/2 pathway, resulting in reduced cell proliferation [73].
		Melittin inhibits sCLU, inactivating both the cholesterol/NF-κB/Bcl-2 axis and the cholesterol/p-ERK axis to suppress tumor growth in pancreatic cancer [74].
		sCLU exhibits an antiproliferative property by inactivating the TAK1/NF-κB axis, preventing the transforming growth factor beta receptor 1 (TGFBR1) from recruiting the TNF receptor-activating factor 6 (TRAF6)/TAK1-binding protein 2 (TAB2)/TGF-β-activated kinase 1 (TAK1) complex to inhibit tumor proliferation and growth in human non-small cell lung cancer (NSCLC) [75].
		Metformin, a conventional medication for type II diabetes, exhibits antitumor effects. Metformin suppresses sCLU, thereby hindering tumor growth through the inactivation of the SREBP-1c/fatty acid synthase (FASN) axis [76].
		sCLU promotes cell growth and proliferation by upregulating the expression of the calcium-binding protein S100A4 in renal cancer [77].
Chemoresistance and Chemosensitivity		The stress response induced by treatments such as radiotherapy and chemotherapy lead to the overexpression of sCLU, a cytoprotective chaperone, which, by binding to activated Bax, it impedes the discharge of cytochrome c and prevents apoptosis [78].
Epithelial–Mesenchymal Transition and Metastasis		In nasopharyngeal carcinoma, CLU undergoes positive regulation by N, N′-dinitrosopiperazine (DNP), triggering MMP-9 and VEGF expression, thus facilitating to metastasis [79].
		In breast cancer, CLU collaborates with eHsp90α to activate key signaling pathways, promoting EMT, migration, and tumor metastasis [80].
		In colon cancer, CLU interaction with platelets activates the p38MAPK pathway and positively regulates MMP-9, facilitating invasion [81].
		Studies on prostate cancer it has been demonstrated that miRNA-217-5p exerts control over the processes of invasion and migration by specifically targeting CLU [82].

**Table 2:** Clusterin as a potential biomarker in various types of cancer.

Types of Cancer	Expression of CLU In Vitro	Expression of CLU In Vivo
Non-small cell lung	Non-small cell lung cancer cell lines show overexpression upon treatment with chemotherapy or radiotherapy. ASO therapy sensitizes cells to these treatments and decreases their metastatic potential [103]	Patients exhibiting positive CLU expression tend to experience improved overall disease-free survival compared to those with negative CLU expression [104].
		More than 80% of the tumors are immunoreactive for CLU [104].
Gastric		Overexpression of sCLU correlates significantly with metastasis, tumor invasion, and TNM stage. In addition, it correlates with unfavorable survival for advanced-stage gastric cancers [105].
Ovarian		Elevated sCLU levels show an inverse relationship with the tumor apoptotic index and are detected more frequently in metastatic lesions than in primary tumors [106].
		Increased sCLU expression is associated with increased biological aggressiveness and decreased survival [107].
Endometrial		When CLU is expressed in endometrial tumors, it is associated with a lower stage, supporting its role in the diagnosis of endometrial carcinoma [108].
		There has been detected higher mRNA expression in both neoplastic and hyperplastic tissues compared to a normal endometrium. In this regard, an increase in mRNA expression of the specific sCLU isoform has been observed in neoplastic and hyperplastic endometrial diseases, but an increase in CLU protein has not been detected. Furthermore, specific CLU immunoreactivity has been observed in all glandular cells of the endometrium compared to other cellular compartments where CLU immunoreactivity was lower or absent [109].
		Increased CLU expression enhances paclitaxel resistance in endometrial cancer [110,111].
Breast	Studies with the MDA-MB-231 cell line show how sCLU silencing significantly inhibits cell proliferation and drastically reduces cell invasion, cell progression and metastatic potential [112,113].	Unlike benign lesions, atypical hyperplasias, intraductal carcinomas, and invasive carcinomas are characterized by CLU overexpression [114].
		Overexpression of sCLU is observed in a higher percentage of triple-negative breast cancer [115] and is associated with a negative estrogen and progesterone receptor status [114].
		Likewise, overexpression in the stroma tends to directly correlate with resistance to preoperative neoadjuvant chemotherapy in the primary tumor and inversely with the apoptosis rate, indicating that gene expression may not be necessary for apoptotic cell death [114,116,117].
Colon		sCLU is overexpressed, while nCLU is downregulated [118]. Likewise, increased sCLU expression was predominantly observed in the cytoplasm of highly invasive tumors and metastatic lymph nodes [31], indicating that CLU expression might serve as a marker to identify patients with more aggressive tumors who could potentially benefit from targeted treatments [119].
Bladder	Treatment with the antisense oligonucleotide (ASO) targeting negative regulation of Bcl-2 and CLU increases the sensitivity of partially resistant bladder carcinoma cells to the tumor necrosis factor-related apoptosis-inducing ligand (TRAIL) [120].	The recurrence-free survival time of patients with overexpression of CLU was shorter than that of patients with normal CLU expression [121,122].
Hepatocellular		High levels of sCLU are associated with migration, invasion, and metastasis [15] due to increased MMP-2 expression and decreased E-cadherin expression [123].
		Furthermore, sCLU overexpression contributes to oxaliplatin resistance [15].
		In peripheral blood mononuclear cells (PBMC) from hepatocellular carcinoma patients, CLU has been proposed as a prospective detection biomarker along with other genes for its sensitivity and specificity [124]. The combination of CLU and AFP further improves diagnostic performance [125].
		The initial levels of CLU are higher for patients with progressive disease than for those with partial or complete response, respectively [126,127].
Pancreatic	The inducer of ferroptosis, a type of cell death characterized by the accumulation of reactive oxygen species (ROS), interferes with apoptotic cell death by regulating CLU [128].	CLU expression in stages I and II is not significantly associated with apoptosis. Moreover, patients with positive CLU expression present better survival rates [129].
Melanomas	Increased expression is linked to heightened drug resistance and extended survival of tumor cells, whereas suppression diminishes resistance and lowers the survival rate of melanoma cells, both in laboratory settings and within living organisms [130].	
Esophageal squamous cells		Elevated CLU expression is associated with unfavorable outcomes in locoregional, overall, and distant progression-free survival. Additionally, individuals exhibiting CLU overexpression in both epithelium and stroma tend to have shorter survival times [131,132].
Head and neck	Overexpression of CLU has been observed, but its implications have not yet been determined [133].	Although CLU is detected in a low proportion of laryngeal carcinomas, it seems to exert a significant role in local invasiveness [133].
Anaplastic large cell lymphomas		The role of CLU is unknown, but its expression within this lymphoma type provides an additional marker for diagnosis [134].
		CLU expression does not correlate with the expression of anaplastic lymphoma kinase-1 (ALK-1). In reactive lymphoid tissues, only fibroblastic reticular cells and follicular dendritic cells exhibit positive expression [134].
Osteosarcoma		sCLU overexpression is associated with metastasis and chemotherapy resistance [135].
Prostate	The expression of CLU increases in advanced stages of cancer, and its suppression sensitizes cells to chemotherapeutic drugs [136].	It has been observed that CLU expression decreases considerably compared to benign tissues [137].
Renal	The introduction of the CLU gene enhances the metastatic potential of renal cell cancer [138], while the removal of the CLU gene inhibits growth and migration [139].	
